# Antioxidant, Antimicrobial, and Insecticidal Properties of Chemically Characterized Essential Oils Extracted from *Mentha longifolia*: In Vitro and In Silico Analysis

**DOI:** 10.3390/plants12213783

**Published:** 2023-11-06

**Authors:** Meryem Tourabi, Ghizlane Nouioura, Hanane Touijer, Asmae Baghouz, Asmae El Ghouizi, Mohamed Chebaibi, Meryem Bakour, Driss Ousaaid, Khalid S. Almaary, Hiba-Allah Nafidi, Mohammed Bourhia, Khallouki Farid, Badiaa Lyoussi, Elhoussine Derwich

**Affiliations:** 1 Laboratory of Natural Substances, Pharmacology, Environment, Modeling, Health & Quality of Life, Faculty of Sciences, Sidi Mohamed Ben Abdellah University, Fez 30003, Morocco; meryem.tourabi@usmba.ac.ma (M.T.); gnouioura@outlook.fr (G.N.); asmae.elghouizi@usmba.ac.ma (A.E.G.); meryem.bakour@usmba.ac.ma (M.B.); driss.ousaaid@usmba.ac.ma (D.O.); lyoussi@gmail.com (B.L.); elhoussinederwich@yahoo.fr (E.D.); 2 Laboratory of Biotechnology, Environment, Agri-Food and Health, Faculty of Sciences Dhar El Mahraz, Sidi Mohamed Ben Abdellah University, Fez 30003, Morocco; hananetou@gmail.com; 3 Laboratory of Biotechnology, Conservation, and Valorization of Natural Resources, Department of Biology, Faculty of Science Dhar El Mahraz, University of Sidi Mohamed Ben Abdellah, B.P. 1796 Atlas, Fez 30003, Morocco; asmae_bio91@yahoo.fr; 4 Ministry of Health and Social Protection, Higher Institute of Nursing Professions and Health Techniques, Fez 30000, Morocco; mohamed.chebaibi@usmba.ac.ma; 5 Biomedical and Translational Research Laboratory, Faculty of Medicine and Pharmacy of Fez, Sidi Mohamed Ben Abdellah University, Fez 30000, Morocco; 6 Department of Botany and Microbiology, College of Science, King Saud University, P.O. Box 2455, Riyadh 11451, Saudi Arabia; kalmaary@ksu.edu.sa; 7 Department of Food Science, Faculty of Agricultural and Food Sciences, Laval University, Quebec City, QC G1V 0A6, Canada; hiba-allah.nafidi.1@ulaval.ca; 8 Department of Chemistry and Biochemistry, Faculty of Medicine and Pharmacy, Ibn Zohr University, Laayoune 70000, Morocco; 9 Ethnopharmacology and Pharmacognosy Team, Department of Biology, Moulay Ismail University of Meknes, Errachidia 52000, Morocco; f.khallouki@fste.umi.ac.ma; 10 Unity of GC/MS, GC-FID, City of Innovation, Sidi Mohamed bin Abdellah University, Fez 30003, Morocco

**Keywords:** *Mentha longifolia*, antioxidant activity, antimicrobial activity, insecticidal activity, essential oil, molecular docking

## Abstract

The present study aimed to explore the phytochemical profile, and evaluate the antioxidant, antimicrobial, and insecticidal properties, of Moroccan *Mentha longifolia* L. essential oil (ML-EO) using in vitro and in silico assays. Noteworthily, as chromatography (GC-MS/MS) revealed that ML-EO is majorly composed of piperitenone oxide (53.43%), caryophyllene (20.02%), and (−) germacrene D (16.53%). It possesses excellent antioxidant activity with an IC_50_ of 1.49 ± 0.00 for DPPH and 0.051 ± 0.06 μg/mL for ABTS. Moreover, the RP and TAC activities were 0.80 ± 0.01 μg/mL and 315.532 ± 0.00 mg EAA/g, respectively. ML-EO exhibited a potent antimicrobial effect, specifically against *Pseudomonas aeruginosa*. It also exhibited strong antifungal ability, especially against *Candida albicans*. Regarding insecticidal activity, for ML-EO, a dose of 20 µL/mL produced a complete reduction in fecundity, fertility, and emergence of adult *C. maculatus* with mortality rates reaching 100%. In silico results showed that the antioxidant activity is mostly attributed to α-Cadinol, the antibacterial efficiency is attributed to piperitenone oxide, and antifungal capacity is related to cis-Muurola-4(15),5-diene and piperitenone oxide. Accordingly, ML-EO has high potential to be used as an alternative for preserving food and stored grain and protecting them against microbes and insect pests in the food and pharmaceutical sectors.

## 1. Introduction

Aromatic medicinal plants (AMP) have a long medicinal history and are still used as an important health primary care of several populations worldwide [1]. They have a large spectrum of biological properties attributed to their dense chemical composition highly affected by environmental conditions [2]. Medicinal plants possess biological activities, including antioxidant, antimicrobial, antidiabetic, anti-obesity, anti-inflammatory effects, and so on [3].

Among medicinal plants, mint (*Mentha* species) belongs to the Lamiaceae (Labiatae) family and comprises 25 to 30 species. It grows in the moderate regions of Africa, Europe, Australia, Asia, and North America [4]. *Mentha longifolia* L. or Himalayan silver mint is well-known in Morocco by its vernacular name “naana touil”, and is considered a perennial plant belonging to the genus *Mentha*. The herb is widely used in folkloric medicine of different countries, including Iraq, Iran, Pakistan, Turkey, and Arab countries as a natural remedy for several diseases, such as gastrointestinal diseases (gas expeller, indigestion, intestinal colic, intestinal ulcer, antidiarrheal, gut spasm, stomach problems, ulcerative colitis) [5], respiratory disorders, (asthma, colds, bronchitis, tuberculosis, sinusitis, and cough) [6] infectious diseases, inflammatory diseases, and menstrual disorders [7]. Furthermore, the plant has considerable economic importance as natural raw matter for the food and pharmaceutical industries [8]. Robust scientific evidence has confirmed multiple pharmacological properties of *M. longifolia* including antihemolytic, anti-inflammatory, antibacterial, hepatoprotective, anti-cancer, and gastroprotective effects [9]. In addition, *M. longifolia* is part of a considerable inventory of essential oils with many therapeutic applications, such as allelopathic properties [10]. Prior research has indicated that the *M. longifolia* essential oil is a potent free-radical scavenger and an effective antimicrobial agent against a broad range of pathogenic microbes [11]. The delve into the phytochemistry of *M. longifolia* revealed the existence of several active biomolecules, including chlorogenic acid, catechin, gallic acid, naringenin, ellagic acid, rutin, daidzein, cinnamic acid, and hesperidin [12]. Ghoulami et al. found that piperitenone oxide and piperitone oxide were characteristic bioactive compounds of *M. longifolia* EO [13]. The phytochemistry of plants is highly affected by several factors, such as ecological ones. Unfortunately, few researchers have evaluated the effectiveness of Moroccan *M. longifolia* EO in fighting nosocomial pathogens and drug-resistant bacteria.

To the best of our knowledge, there is no sufficient research that studied the chemical constituent and in vitro capabilities of EO isolated from *M. longifolia* growing in Morocco. Thus, we aimed to investigate the volatile profile of Moroccan *M. longifolia* EO using GC-MS/MS and study its antioxidant, antimicrobial, and insecticidal activities by use of in vitro and in silico assays.

## 2. Results and Discussion

### 2.1. Volatile Profile of Essential Oil

**Figure 1:**
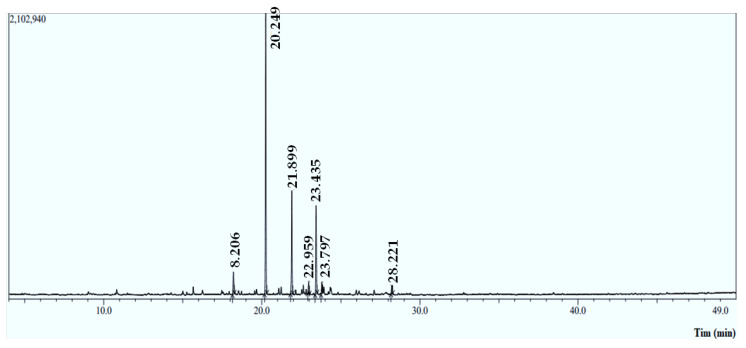
Chromatographic profile of the essential oil (EO) extracted from *M. longifolia* leaves using GC-MS/MS.

**Figure 2:**
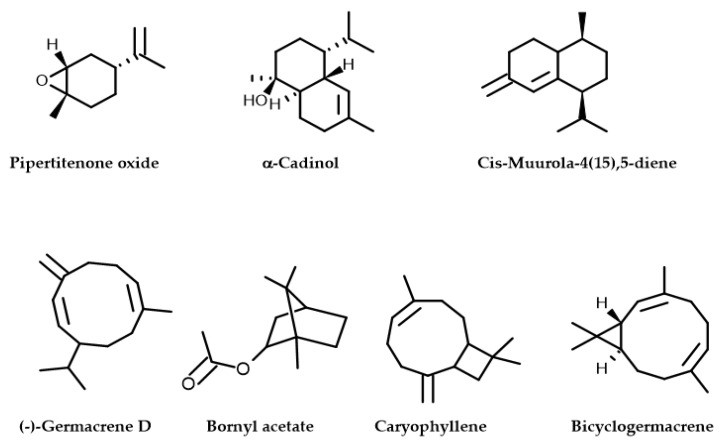
Molecular structures of phytochemical composition in *M. longifolia* EO.

The *Mentha longifolia* EO yield was approximately 0.79 ± 0.265% (*v*/*w*), with seven compounds identified using an HP-5MS capillary column. The Gas Chromatographic examination indicates that the monoterpenoids compounds (Figure 1 and Table 1) predominated in the *M. longifolia* EO, including piperitenone oxide (53.43%) and bornyl acetate (4.47%), followed by the chemical class of sesquiterpene hydrocarbons, which are caryophyllene (20.02%) and (−) germacrene D (16.53%) (Figure 2). These results are in line with those reported by previous studies [14]. In the same context, *M. longifolia* from Brazil was discovered to be rich in piperitenone oxide (60.79%) [14]. Indian *M. longifolia* was shown to be also abundant in piperitenone oxide (54.23%) and trans-piperitone oxide (24.06%) [15].

The variability in the chemical constituents of EO depends on environmental factors. Indeed, the chemical composition of *M. longifolia* oils may change based on the used part of the plant, the season of the vegetative cycle, the maturity of the plant, the harvesting period, and even plant genetic characteristics [16].

The phytoconstituents of EOs, such as monoterpenes hydrocarbons (MH), oxygenated monoterpenoids, and sesquiterpenes hydrocarbons from various medicinal herbs have been identified to possess significant biological and pharmacological capacity, including antioxidant, antimicrobial, insecticidal, and cytotoxic potential [9].

### 2.2. Antioxidant Activity

The antioxidant ability of *M. longifolia* EO was assessed using DPPH, ABTS, RP, and TAC tests and results are summarized in Table 2. A potent antioxidant activity was obtained to scavenge the ABTS•+ radical cation (Table 2). As shown in Table 2, the EO of *M. longifolia* exhibited maximum antioxidant capacity with IC_50_ values of 1.49 ± 0.00 µg/mL for the DPPH test and 0.051 ± 0.06 µg/mL for the ABTS assay compared to BHT and Trolox as a standard (42 ± 0.01 and 24.14 ± 0.19 µg/mL), respectively.

Our data were higher than those reported by Barros and Z. Bouassida and coworkers, who reported IC_50_ values were 0.86 ± 0.01 mg/mL and 100 µg/mL for their *M. longifolia* essential oil, respectively [17].

To the best of our knowledge, and based on the literature studies, *M. longifolia* antioxidant ability has not yet been reported using the ABTS assay; however, a few studies have been carried out to assess its antioxidant capacity using different test methods, including DPPH and reducing power (RP). The antioxidant ability of ML-EO from Yugoslavia was examined by DPPH scavenging capacity assay [18], a DPPH test used in Saudi Arabia, Bosnia, and Herzegovina that displays potent activity for ML-EO [19].

Regarding the reducing power (RP) of the studied EO, the results are represented in (Table 2) and indicate that the EC_50_ value is relatively high (0.80 ± 0.01 µg/mL) in comparison to the ascorbic acid as standard (31 ± 0.07 µg/mL). Our results are significantly more important than those reported by Bardaweel et al. for Algerian *Mentha spicata* L. with an EC_50_ of 215 ± 4.50 µg/mL [20].

The ammonium phosphomolybdate assay was achieved to determine the total antioxidant capacity of our *M. longifolia* EO. Results were represented in (Table 2) and showed that our EO had a TAC value of 315.53 ± 0.01 mg AAE/mL of EO. These results are stronger than those reported by Mhiri et al. for Tunisian *Mentha piperita*, where the TAC value was 177.00 ± 0.30 mg/g of DW. Another study made by Salamatullah et al. for the EO of *Mentha rotundifolia* var demonstrated that their TAC value was 697.45 ± 1.07 mg AAE/g [21], these findings were higher than those obtained for our essential oil.

Indeed, a previous study of the chemical constitution of *M. longifolia* EO revealed that these EOs contained a wide spectrum of monoterpenoids such as piperitone, piperitenone oxide, carvone, pulegone, β-caryophyllene, menthol, 1,8-cineole, menthone, and limonene [22]. However, several naturally occurring substances such as piperitenone oxide and germacrene D might have contributed to the strong antioxidant capacity [23].

### 2.3. Antibacterial Activities of M. longifolia Essential Oil

The antibacterial potency of *M. longifolia* EO and the minimal inhibitory concentration against five microbe species are shown in Table 3. The EO derived from *M. longifolia* leaves showed significant antibacterial activities depending on the concentration and the antibiotic used (streptomycin sulfate or erythromycin). *M. longifolia* EO showed the strongest antibacterial effect against *Pseudomonas aeruginosa*, with an inhibition diameter of 24.50 ± 0.71 mm and an MIC of 0.005 ± 0.00 µg/mL. In contrast, showed an inhibition diameter of 16.00 ± 1.41 mm and an MIC of 0.015 ± 0.007 µg/mL against *Bacillus subtilis*, an inhibition diameter of 7.55 ± 0.64 and an MIC of 0.010 ± 0.01 against *Escherichia coils*, and an inhibition diameter of 7.50 ± 0.71 mm and an MIC of 0.011 ± 0.00 µg/mL against *Staphylococcus aureus*.

Our data found that the investigated bacterial strains, whether Gram-negative or Gram-positive, were absolutely resistant to Erythromycin and moderately resistant to Streptomycin. These findings are consistent with previous studies, which demonstrated that the most dangerous drug-resistant microbes, were *S. aureus*, *P. aeruginosa*, *E. coli*, *K. pneumoniae*, *A. baumannii*, and *Enterobacter* spp. strains [24].

Our findings demonstrated that Gram-positive and Gram-negative bacteria were most sensitive to the studied EO, which was consistent with the results found by Hajlaoui et al. who discovered that their *M. longifolia* EO had the same positive effect against all tested Gram (−) and Gram (+) pathogens [25]. In the present study, the examined EO revealed a stronger antibacterial effect against *Pseudomonas aeruginosa* with an inhibition diameter of 24.50 ± 0.71 mm. These results were in line with those of Niki and co-workers, which revealed that their *M. longifolia* essential oil has a considerable antibacterial effect against *Pseudomonas aeruginosa* (ATCC 9027), with an inhibition zone of 25 mm [26]. In addition, the *M. longifolia* folium EO gathered in Bosnia and Herzegovina also demonstrated important antibacterial activity against *Escherichia coli* (ATCC 8739) and *Salmonella enterica*. ABONY subsp. enterica (NCTC 6017) with inhibitory diameters ranging from 7 ± 0.33 to 19 ± 0.71 mm and 10 ± 0.99 to 20 ± 0.33 mm, respectively [26]. Furthermore, our *M. longifolia* EO also indicated a high growth inhibition of Gram-positive bacteria particularly *Bacillus subtilis*, with an inhibition diameter of 16.00 ± 1.41 mm. Our results contrasted those found by Gulluce et al. who showed a slight inhibition of 8 mm of *Bacillus subtilis* growth by *Mentha longifolia* L. EO. Gulluce et al. suggested that the shown effect was correlated to the high concentration of cis-piperine epoxide (18.4%), pulegone (15.5%), and piperitenone oxide (14.7%) [27]. In our study, the bacteria *P. aeruginosa* and *E. coli* were shown to be resistant to the antibiotics streptomycin and erythromycin. This resistance can be explained by the barrier effect of these species, where the membrane of Gram-negative bacteria is more complex with a smaller peptidoglycan layer and an outer membrane comprised of a double layer of phospholipids, which block antibiotic diffusion into the cells [28].

Previous reports have described several mechanisms of action of EO antimicrobial effects, including penetrating the bacterial cell wall, cytoplasmic membrane proteins attributed to their lipophilic nature causing ions loss, membrane potential decrease, the collapse of the proton pump, and ATP pool overexploitation [29]. As a result, they induce lipid partitioning of bacterial cell membranes and mitochondria, causing cell lysis and release of cell contents. Altered bacterial enzymatic systems may be a possible antibacterial mechanism [30].

Indeed, the mechanisms of action of EOs depend on their chemical compositions, concentration, structure, aromatic nuclei, and placement of functional groups [31]. The *M. longifolia* EO is high in terpene compounds, particularly oxygenated monoterpenes such as piperitenone oxide and oxygenated sesquiterpenes such as caryophyllene and (−) germacrene D, which are now widely known to have high antimicrobial properties against a variety of microorganism strains [32]. Furthermore, the antibacterial mechanism of action of terpene is not yet clear, our findings indicated that lipophilia, terpene hydrophobicity, and the existence of hydroxyl groups (-OH) in the constituents all play significant roles in the mechanism of antibacterial activity [33].

In the same context, our result indicates that terpenes, a major category in our *M. longifolia* EO, are now widely recognized to show strong antimicrobial activity, which our research also confirmed.

### 2.4. Antifungal Activities of M. longifolia Essential Oil

The disc diffusion approach was used to test the antifungal capacity of our EO against the yeast and fungus *Aspergillus niger* (MTCC 282) and *Candida albicans* (ATCC 10231). Results of the antifungal activity are represented in (Table 3) and indicate that *M. longifolia* EO has a strong effect against both tested strains. The results demonstrate that a high EO inhibition was found for *C. albicans* with an inhibition zone of 20.00 ± 0.00 mm and MIC values of 0.005 ± 0.00 mg/mL, showing a noticeable effect against *A. niger* with a percent of inhibition of 1.50 ± 0.00% and an MIC value of 0.005 ± 0.00 mg/mL (Table 4).

Yeast infections are very frequent among patients in hospitals across the world, and there are several risk factors linked with poor outcomes. Multiple epidemiological studies on fungal infections have displayed that *Candida* is responsible for a wide range of disorders. Additionally, the fourth highest frequent source of nosocomial bloodstream infection is a Candida fungus, computing for 8–10% of infections [34]. The *Aspergillus* spp. can induce invasive infections, causing mortality and serious diseases in various types of patients, including those in serious attention units [35].

Findings indicate that our EO has a potent antifungal capacity against *C. albicans* with an inhibition zone of 20.00 ± 0.01, more than *A. niger* with an inhibition percentage of 1.50 ± 0.02 compared to an antibiotic (fluconazole). The result is consistent with those reported by Mkaddem and coworkers, which indicated that the EO of *M. longifolia* collected from Tunisia revealed antifungal efficiency against *Candida albicans* (IPA 200) with an inhibition diameter of 19 mm [36]. The EO of *M. longifolia* collected from Saudi Arabia showed moderate antifungal ability against *A. niger* with a percent inhibition ranging between 2.2 and 3.3% [19]. Similarly, the findings of another study showed notable antifungal activity, especially against *Aspergillus flavus*, *Aspergillus fumigatus*, *Aspergillus niger*, and *Fusarium culmorum* with 100% fungal mycelial inhibition development at the concentration of 500 µL/mL [37].

The volatile profile of *M. longifolia* EO could express its antifungal activity [26]. The antifungal effect of *M. longifolia* essential oil may be attributed mostly to its chemical components; it is particularly rich in piperitenone oxide, which is widely recognized for its antimicrobial activity, especially its antifungal activity [38].

According to several studies, the toxicity impact of EO and their derivatives is associated with their capacity to block or inactivate fungal cell walls and cell membrane, coagulate the cytoplasm, consequently damage cell components, and allow macromolecules to leave [39]. The hydrophobic components in EO could affect the permeability of microbial cell membranes to cations such as H+ and K+, altering the passage of protons, controlling cellular pH, and affecting the chemical structure and function of the cells [40].

### 2.5. Insecticidal Activity

In many countries of the globe, it is an ancient tradition to apply natural sources of plant materials, specifically focusing on EOs, to protect agricultural products from various insect pests. *Callosobruchus maculatus* is one of the most significant storage insect pests of cowpea grains [41].

In the present work, we examined the toxic potential of *M. longifolia* EO against *C. maculatus* over 24 h at different doses using fumigation and repellency tests. The lethal concentration (LC_50_) was also computed for each EO concentration at the appropriate treatment durations.

### 2.6. Fumigation Bioassay

#### 2.6.1. Effect of ML-EO on Adult Mortality

The insecticide efficiency of ML-EO is represented in (Table 5). Our finding demonstrated that the fumigation bioassay variability is due to the oil doses, plant species, and exposition period. ML-EO is more toxic in all doses tested, showing 100% mortality against *C. maculatus* at doses of 12.0 and 20.0 μL/L after 18 h of treatment. The lethal dose (LC_50_) for *M. longifolia* essential oil was 29.42 and 2.24 µL/L in air at 3 h and 12 h postexposure, with respective 95 percent confidence intervals (19.96–309.41; 0.0–4.68). In contrast, the LC_90_ ranged from 73.09 to 18.74 µL/L air (Table 6). According to previous reports, essential oil from *M. longifolia* examined during a fumigation test showed moderate fumigation toxicity against *Sitophilus zeamais* at concentrations of (24 and 32 µL/L in air) [42]. The efficiency of ML-EO was in line with the outcome reported by Khani and coworkers, who reported that the ML-EO exhibited potent insecticidal activity against *Callosobruchus maculatus* with an LC_50_ of 13.05 μL/L air [43]. The presence of major monoterpenes and sesquiterpenes notably piperitenone oxide, germacrene D, in the studied EO, could be attributed to their strongest insecticidal activity since this compound had been reported to possess a strong insecticidal efficiency [44].

#### 2.6.2. Effect of ML-EO on Fecundity

**Figure 3:**
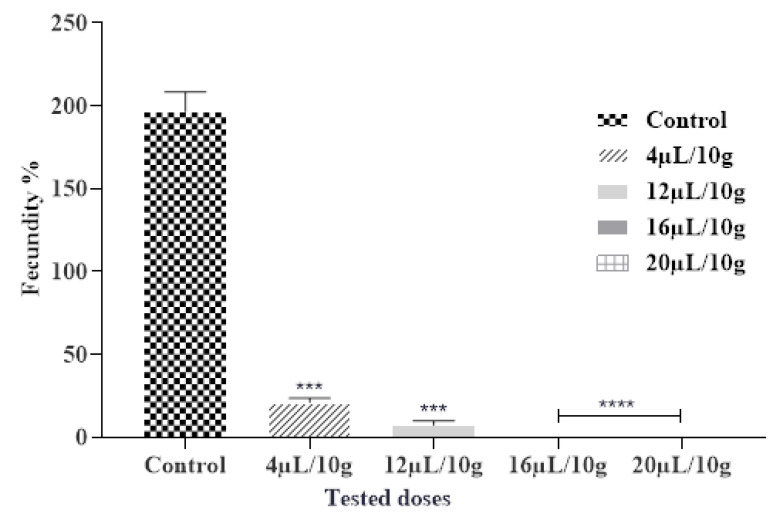
Fecundity % of *C. maculatus* in the fumigation test by the exposure to the ML-EO at different doses (Control, 4, 12, 16, 20 μL/10 g). The outcomes are presented as mean ± SD. (*) comparison between the control group and all remaining concentrations. (The significance initiates at *** *p* < 0.001; **** *p* < 0.0001).

The insecticidal effects of the tested EOs affect significantly the fecundity of *C. maculatus* females. The obtained findings demonstrated a considerable reduction in the number of eggs deposited by females compared to the control after being exposed to the vapor of ML-EO (Figure 3 and Table 7). A dose of 16 µL/L of ML-EO completely reduces the fecundity of female *C. maculatus*. The ML-EO inhibited female fecundity in comparison with control values of 196.66 ± 11.55 at the highest test concentration (20 µL/10 g seeds). Statistical analysis showed that the EO-induced toxicity against *C. maculatus* fecundity was extremely significant for the different concentrations (ANOVA: F = 742.7; df = 4, 10; *p* < 0.0001). Previous research suggested that various plant derivatives, such as EOs and their components, decreased insect fecundity [45]. Based on the literature researchers, this is the first study on the efficiency of ML-EO on the fecundity of *C. maculatus* grain cowpea pest. In contrast, the efficiency of essential oils of *Mentha* species on fecundity had been studied against various pests, including the effect of *M. × piperita* L. essential oil on reduction in fecundity of *Acanthoscelides obtectus*, reduction in fecundity/female/day was 3.1 ± 2.0 [46]. *M. pulegium* L. essential oil reduces 82–88% in female fecundity of *C. maculates* at a concentration of (0.25–1%) [47]. In the seam context, Saxena and Mathur hypothesized that the reduced fecundity by the plant derivatives may be related to the disruption of regulatory processes as opposed to a direct impact on the ovarian tissue [48]. Previous reports illustrate that plant EOs have important effects on oogenesis, causing a reduction in oviposition, and appear to have juvenomimetic activity similar to juvenile and molting hormones [49].

#### 2.6.3. Effect on Fertility

**Figure 4:**
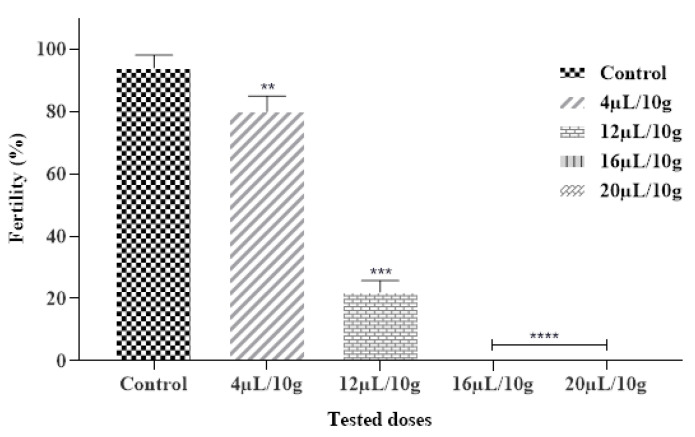
Fertility % of *C. maculatus* in the fumigation test by the exposure to the ML-EO at different doses (Control, 4, 12, 16, 20 μL/10 g). The outcomes are presented as mean ± SD. (*) comparison between the control group and all remaining concentrations. (The significance initiates at: ** *p* < 0.01; *** *p* < 0.001; **** *p* < 0.0001).

Our findings also showed that the tested EO had an inhibitory effect on the fertility of *C. maculatus*. Results obtained expressed that each ML-EO significantly decreased the egg production in a dose and time-dependent approach as compared to the control (Figure 4 and Table 7). In addition, ML-EO exhibited a potent hatching egg (fertility) inhibitory effect at a dose of 16 μL/10 g compared to the control. The insecticidal efficacy of the essential oils examined showed that the fertility of *C. maculatus* females was extremely significant between the different concentrations (F = 548.5; df = 4, 10; *p* < 0.0001). In our study, the lowest used dose of ML-EO, the female *C. maculatus* eggs were prevented from hatching. We can thus deduce that our outcomes were stronger than those reported by Allali and coworkers, especially at a dose of 20 µL/L of essential oil extracted from *M. pulegium* L., which inhibited 100% of the fertility of *C. maculatus* [50].

#### 2.6.4. Effect on Adult Emergence

**Figure 5:**
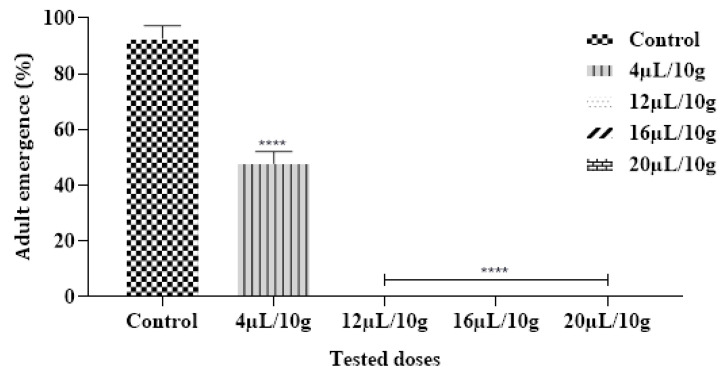
Adult emergence % of *C. maculatus* in the fumigation test by exposure to the ML-EO at different doses (Control, 4, 12, 16, 20 μL/10 g). The outcomes are presented as mean ± SD. (*) comparison between the control group and all remaining concentrations. (The significance starts at **** *p* < 0.0001).

The achieved findings demonstrated that no *C. maculatus* adults emerged in Cowpea Seeds that were provisory treated with ML-EO starting at a concentration of 12 µL/10 g (Figure 5 and Table 7). According to previous research, the EO from *Mentha* species had the greatest effect against a range of insect pests [44]. In contrast, EO extracted from *M. pulegium* L. assessed by inhalation against *C. maculatus* reduced the emergence of larvae with 100% death after 24 h of application with doses of 20.0 µL/L air using contact assay [50].

#### 2.6.5. Repellency Test

The herbal tradition has a great history of using plants as insect repellents, especially aromatic herbs and oils [51]. Various experiments were performed to explore the repellent capabilities of *Mentha* species against agricultural insect pests [42]. In the present study, the repellent effect is dependent on the plant and used concentration. Indeed, ML-EO showed a potent repellent efficiency. In the filter paper disc assay, all concentrations of ML-EO demonstrated greater than 70% repulsion power against *C. maculatus* within 24 h of treatment (Table 8). According to the classification by McDonald, 1970, ML-EO had a strong repulsive power of 100% at the high dose of 20 µL/cm^2^ with an average repulsion percentage of 89.75%. This EO is within the repellant class following McDonald, 1970. The obtained outcome corroborated with those described by Odaymi and coworkers who found that *M. longifolia* EO completely (100%) repelled adult *Sitophilus zeamais* at all concentrations [42].

Several studies have been performed on plant extracts, particularly essential oils of *Mentha* species, to examine their insecticidal activity against pest insects that attack stored green. Our finding demonstrated that the ML-EO was highly efficient against the *Callosobruchus maculatus* insect pest. These outcomes are consistent with those that were previously reported [42].

Likewise, many plant extracts and essential oils contain volatile chemicals that have insecticidal properties and are constituted of, alcohols, alkanes, aldehydes, and terpenoids, particularly monoterpenoids [52]. Essential oils and their constituents affect biochemical processes in various and different pathways, altering the endocrinologic balance of insects in particular [53]. Monoterpenoids and sesquiterpenoids are extremely volatile compounds that defend plants against insect infestations. Monoterpenoids are lipophilic chemicals, that have been studied for their neurotoxic effect. They can produce symptoms such as convulsions, hyperactivity, and tremors succeeded by paralysis similar to the effect of organophosphate and carbamate insecticides [44]. These compounds operate through several modes of action including the modulation of GABA, blocking neurotransmitters such as acetylcholinesterase [54], and the impact on octopamine synapses [55]. Acetylcholinesterase (AChE) inhibition is a specific enzyme for saved substance insect control agents that can block the neurotransmitter acetylcholine in the synaptic gap [54].

The essential oil of *M. longifolia* is rich in diverse chemical compounds, however, some components are present in larger quantities, for example, piperitenone oxide, 1,8-cineole, germacrene D, and caryophyllene. Previous research has shown the insecticidal effects of piperitenone oxide, as well as other terpenes, against a variety of pests [56].

Piperitenone oxide and caryophyllene is the major component in our EO. According to Miyazawa et al. piperitenone oxide has insecticidal and acetylcholinesterase (AChE) inhibitory effects [57]. Consequently, Lee et al. suggested that, in addition to blocking AChE, monoterpenes may also act on other vulnerable sites such as cytochrome P450-dependent monooxygenases [58]. Thus, the insect octopamine receptor pathway is more sensitive to some oils compared to AChE. The essential oil affects octopamine receptors to increase heart rate or cAMP levels in insects [59]. Kavallieratos et al. showed that the mechanism of action of the piperitenone oxide against insects has been performed via the coupling of the exocyclic carbonyl with the conjugated C-C double bond, enabling it to penetrate the cuticle rapidly and reach the target sites [56].

Accordingly, the inhibitory effect of essential oil components was induced by synergistic effects around various chemical groups relatively more than by a single powerful inhibitor.

Overall, this study suggests that essential oils derived from Moroccan *Mentha longifolia* and such species have potential as natural insect repellents for controlling *C. maculatus* invasion in stored cowpea grains. However, further research is needed to determine the optimal concentration and application technique for these essential oils in a practical pest management context.

### 2.7. Molecular Docking

Inhibition of NADPH in antioxidant activity plays a significant role in regulating cellular redox balance and antioxidant defense mechanisms. NADPH (nicotinamide adenine dinucleotide phosphate) is a crucial cofactor in several enzymatic reactions that contribute to cellular antioxidant defenses.

Alpha-cadinol and piperitenone oxide are naturally occurring compounds, commonly found in the essential oils of various plants. These compounds have attracted attention in the field of natural products and health research due to some preliminary evidence suggesting that they may possess antioxidant activity [60,61].

Antioxidants are substances that play a crucial role in protecting cells and tissues from oxidative damage caused by free radicals. Free radicals are highly reactive molecules that can harm biological molecules such DNA, proteins, and lipids potentially contributing to aging and various diseases.

The antioxidant activity of alpha-cadinol and piperitenone oxide is thought to be linked to their ability to scavenge free radicals and neutralize their detrimental effects. Essentially, they act as electron donors, stabilizing free radicals by donating electrons and preventing them from causing cellular damage.

In our in silico study, all the molecules identified in the bulk of *M. longifolia* EO presented a remarkable antioxidant activity represented by binding energy between −3.585 and −6.041 Kcal/mol. The α-cadinol was the most powerful substance to combat NADPH oxidase with a glide score of −6.041 Kcal/mol followed by piperitenone oxide (−5.195 Kcal/mol) (Table 9).

Regarding antimicrobial activity, several studies on the essential oils of plants, in which alpha-cadinol and piperitenone oxide are the major compounds, have shown remarkable antibacterial activity [62,63].

Piperitenone oxide was the most active molecule against *E. coli* with a glide score of −7.104 Kcal/mol. While α-Cadinol is the most active against *S. aureus* with a glide score of −5.714 Kcal/mol.

Concerning the antifungal ability, cis-Muurola-4(15),5-diene showed strong capacity against *Candida albicans* with a glide score of −7.486 Kcal/mol. While piperitenone oxide was the most effective chemical against *Aspergillus niger* with a glide score of −4.687 Kcal/mol (Table 9).

**Figure 6:**
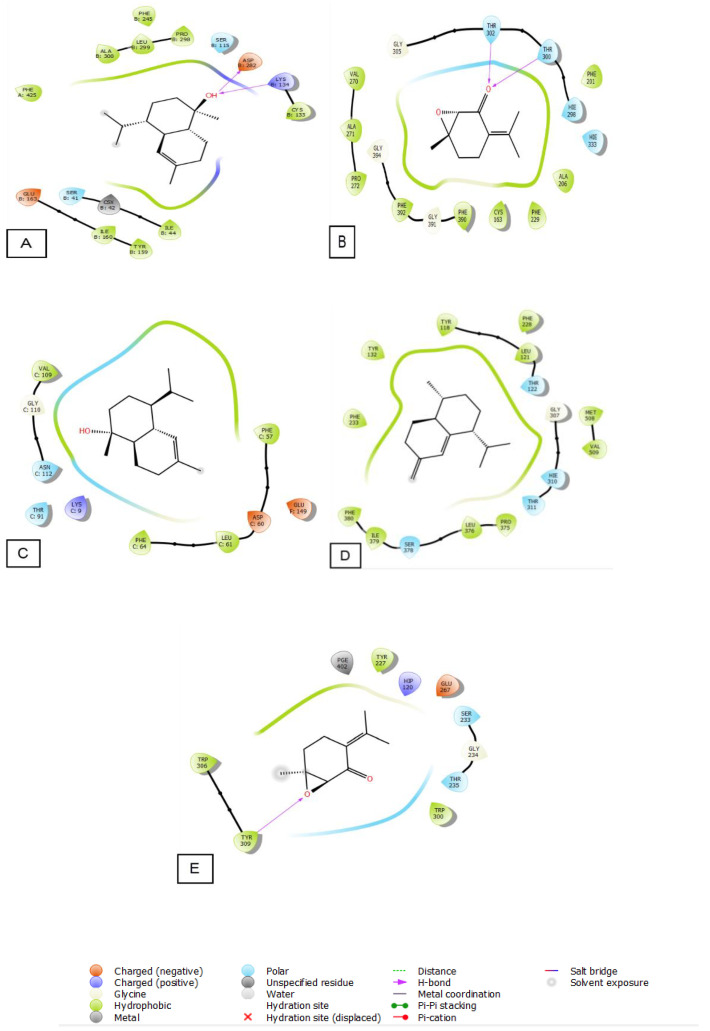
The 2D viewer of ligands interactions with the active site. (**A**): α-Cadinol interactions with the active site of NADPH oxidase. (**B**): piperitenone oxide interactions with the energetic site of beta-ketoacyl-[acyl carrier protein] synthase from *Escherichia coli.* (**C**): α-Cadinol interactions with the active site of *Staphylococcus aureus* nucleoside diphosphate kinase. (**D**): cis-Muurola-4(15),5-diene interactions with the active site of sterol 14-alpha demethylase (CYP51) from a pathogenic yeast *Candida albicans*. (**E**): piperitenone oxide interactions with the energetic site of a beta-1,4-endoglucanase from *Aspergillus niger*.

**Figure 7:**
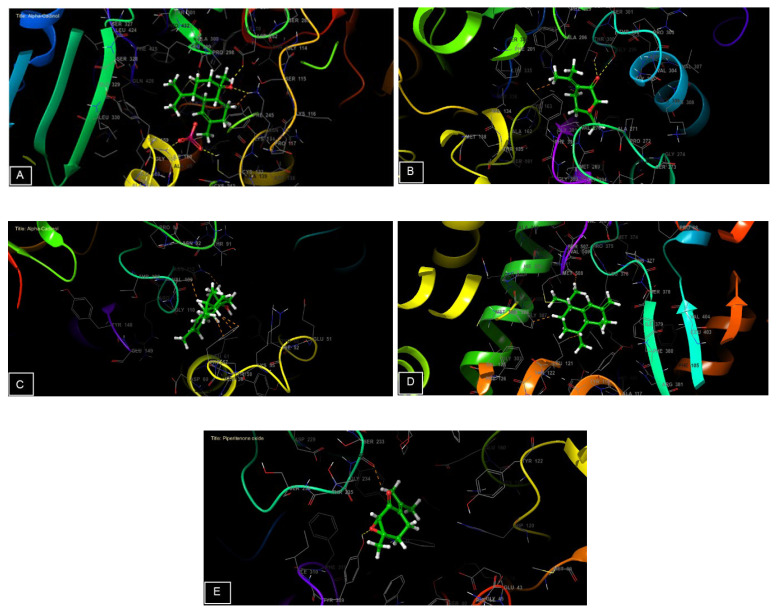
The 3D viewer of ligands interactions with the active site. (**A**,**C**): α-cadinol interactions with the active site of NADPH oxidase and *Staphylococcus aureus* nucleoside diphosphate kinase. (**B**,**E**): piperitenone oxide interactions with the active site of beta-ketoacyl- [acyl carrier protein] synthase from *Escherichia coli* and a beta-1,4-endoglucanase from *Aspergillus niger*. (**D**): cis-Muurola-4(15),5-diene interactions with the active site of sterol 14-alpha demethylase (CYP51) from a pathogenic yeast *Candida albicans*.

The 2D and 3D visualization of the interaction between the active site and ligands of NADPH oxidase has shown that α-Cadinol is capable of forming two hydrogen bonds with residues of ASP 282 and LYS 134. In the energetic site of beta-ketoacyl-[acyl carrier protein] synthase from *Escherichia coli*, piperitenone oxide has established two hydrogen bonds with residues of THR 300 and THR 302. Additionally, piperitenone oxide has formed a single hydrogen bond with residue TYR 309 in the energetic site of beta-1,4-endoglucanase from *Aspergillus niger* (Figure 6 and Figure 7).

### 2.8. Statistical Analysis

#### Principal Component Analysis

**Figure 8:**
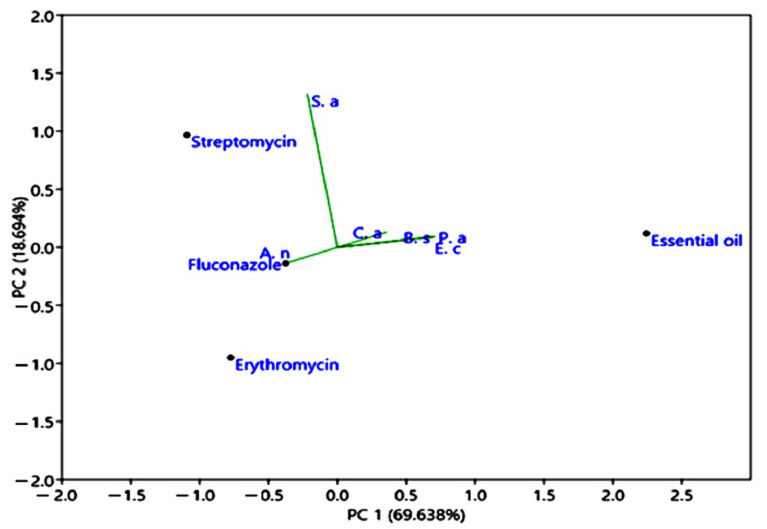
Principal component analysis (PCA), presents the correlations of antimicrobial properties of *M. longifolia* EO compared to erythromycin, streptomycin, and fluconazole. *Ca*: *Candida albicans*; *Sa*: *Staphylococcus aureus*; *Ec*: *Escherichia coli*; *Bs*: *Bacillus subtilis*; *An*: *Aspergillus niger*; *Pa: Pseudomonas aeruginosa*.

The previously acquired results were treated using the principal component analysis (PCA), and the key outputs are displayed in Figure 8. Notably, principal component analysis is a dimensionality-reduction method that is used to condense a large set of variables into a more manageable set while retaining the majority of the details in the larger set.

The principal component analysis of *M. longifolia* EO was based on the correlations of antimicrobial activities of *M. longifolia* compared to standard medicine (erythromycin, streptomycin, and fluconazole). The first component explained (69.638%), and represented, in its positive part, the inhibition diameter values of *Pseudomonas aeruginosa*, *Bacillus subtilis*, *Candida albicans*, and *Escherichia coli* and, in its negative part, the inhibition diameters values of *Staphylococcus aureus* and *Aspergillus niger*. The second component explained (18.694%) and represented, in the positive part, the inhibition diameter values of *Candida albicans*, *Staphylococcus aureus*, *Escherichia coli*, *Bacillus subtilis*, and *Pseudomonas aeruginosa*. Whereas in the negative part, we found only the diameter inhibition value of *Aspergillus niger*.

In addition, Figure 8 showed similar sensitivity to *M. longifolia* essential oil for the following strains: *Candida albicans*, *Escherichia coli*, *Bacillus subtilis*, and *Pseudomonas aeruginosa*. Similar outcomes were shown in another study carried out by Lafraxo et al. [64].

It has been shown that the hydrocarbons and oxygenated monoterpenes found in *Mentha longifolia* essential oil are able to damage the cellular integrity of bacteria, thus impeding respiration and ion transport functions [65]. In addition, it was observed that Gram-positive bacteria are slightly more resistant to the studied essential oil than Gram-negative bacteria. This can be explained by the difference in the bacterial wall composition [66].

## 3. Material and Methods

### 3.1. Collection of Plant Materials

Aerial elements of *Mentha longifolia* were collected from the town of Ifran (Morocco’s Middle Atlas Mountains, latitude 33°31′35″ N; longitude 5°06′ 36″; altitude 1648 m) in June 2021, and it was identified by Professor Amina BARI the botanist of the department of biology, at the Sidi Mohamed Ben Abdellah University. The voucher specimen has been deposited at the faculty herbarium under number: 001MLAV202162. The collected plant was dried in dark conditions for two weeks, and then the aerial parts were removed and powdered using a professional herb grinder. The obtained powder was packed into a glass bottle and stored in a dark and heat-protected place until extraction.

#### 3.1.1. Extraction of the Volatile Oils

The dry (flowering) leaves of *M. longifolia*, (100 g) were slightly ground, the plant materials were placed in a 2 L flask with 1 L of distilled water, and the combination was heated for 3 h while refluxing using a Clevenger-type apparatus [67]. The EO obtained was separated from water with anhydrous sodium sulfate, stored in a closed glass flask, and kept at 4 °C in the dark conditions until the in vitro test. At leas three separate extractions were performed, and the mean yield and standard error were calculated.

#### 3.1.2. Chemical Characterization of Essential Oil by GC/MS/MS

Gas chromatography with an ion trap mass spectrometry apparatus was used for the GC-MS analysis (Trace GC ULTRA S/N 20062969/Polaris Q Thermo Fischer, Waltham, MA, USA). For the chromatographic separations, an HP-5MS capillary column (60 m × 0.32 mm; coating thickness 0.25 μm) was employed. Scan range: 40–650 amu; scan rate: 3.9 scans/s; temperatures of the transfer line and ionic source: 300 °C and 200 °C, respectively. The oven temperature was programmed to increase from 40 to 280 °C in a 5 °C/min range; the injector temperature was 260 °C; helium was used as the carrier gas at 1 mL/min; and 1 µL of a 10 percent cyclohexane essential oil solution was injected; the split ratio was 1:30. The retention times were compared to those of authentic samples, their indices of linear retention were compared to the series to (C8–C29) alkanes, and computer matching against commercial (NISTMS) and laboratory-developed library mass spectra built up from pure substances and components of known oils and MS literature data were used to determine the components identification [68].

### 3.2. Antioxidant Ability of Essential Oil

#### 3.2.1. DPPH Scavenging Capacity

The scavenging capacity of free radicals in EO was performed by the scavenging method of 1,1-diphenyl-2-picrylhydrazyl (DPPH) reported by Burits and Bucar [69]. Briefly, 50 µL of EO dissolved in ethanol was used with different concentrations. A total of 825 µL of DPPH solution (60 μM) and an absorbance of 0.7 at 517 nm) was added to every dilution. The absorbance measurements at 517 nm wavelength were conducted after incubation for 60 min at room temperature. The IC_50_ values were determined from the graph by the percentage of inhibition (*PI*), according to the following formula.

PI (%)=[(Abscontrol−Abssample|Abscontrol)×100]

BHT was chosen as a positive control and the IC_50_ value was calculated from the percentage of the inhibition curve.

#### 3.2.2. Radical Cation Decolorization (ABTS Assay)

Radical cation (ABTS+) decolorization of the extracts was measured according to the protocol described by RE et al. [70]. For this, 825 μL of 2,2′-casino-bis (3-ethylbenzothiazoline-6-sulfonic acid) diammonium salt (ABTS was dissolved in water to a 7 mM concentration. ABTS radical cation (ABTS•1) was produced by reacting ABTS stock solution with 2.45 mM potassium persulfate (final concentration) and allowing the mixture to stand in dark conditions at room temperature for 12–16 h before use) was added to 50 μL of each essential oil, after 30 min of incubation in the dark, the absorbances were measured at 734 nm using a UV/Vis spectrophotometer. The percentages of ABTS inhibition were calculated according to the following formula:

(1) $$\%inhibition=\left[\left(Ac-As|Ac\right)\times 100\right]$$

The results were calculated in triplicate and the values were represented in mg/mL.

Trolox was chosen as a positive control and the IC_50_ value was calculated by use of the percentage of the inhibition curve.

#### 3.2.3. Reducing Power (RP)

The reducing power of EO has been assessed by the procedure described by Parki et al. [71], with slight modification. Briefly, 250 μL of 0.2 M sodium phosphate buffer (pH 6.6), and 250 μL of 1% potassium ferricyanide were added to 50 μL of *M. longifolia* essential oil, the mixture was incubated at 50 °C for 20 min, then 250 μL of 10% TCA was added and immediately mixed with 250 μL of distilled water and 60 μL of 0.1% ferric chloride. The absorbances were measured at 700 nm with a PerkinElmer Lambda 40 PerkinElmer spectrophotometer.

Ascorbic acid was used as the standard. The results were expressed in EC50 (Half maximal effective concentration), which was calculated by use of the absorbance curve (Y = ax + b; Y = 0.5). The EC_50_ values were represented in μg/mL.

#### 3.2.4. Total Antioxidant Activity (TAC)

The ammonium phosphomolybdate test was used for the assessment of total antioxidant potential (TAC) according to the protocol described by Prieto et al. [72]. Here, 25 μL of EO or standard (Ascorbic acid) was mixed with 1 mL of reagent solution (sulfuric acid 6 M, sodium phosphate 28 mM, and ammonium molybdate 4 mM). After incubation in a heated bath for 90 min at 95 °C, the sample’s absorbance was determined at 700 nm. The sample’s absorbance was determined at 700 nm against the blank with a PerkinElmer Lambda 40 UV/Vis, (Barcelona, Spain) spectrophotometer. The values obtained were represented as milligrams of ascorbic acid equivalent per gram of EO (mg AAE/g EO) and the standard curve (Y = 2.9009x + 0.0999, R^2^ = 0.9991).

### 3.3. Antimicrobial Activities of Essential Oil

The antimicrobial capacity of the EO was examined against five Gram-negative bacteria including *Escherichia coli* (K12) and *Pseudomonas aeruginosa* (CIP A22) and Gram-positive bacteria including *Staphylococcus aureus* (ATCC 6633) and *Bacillus subtilis* (DSM 6333), and against two fungal strains including *Candida albicans*; (ATCC 10231), and *Aspergillus niger* (MTCC 282). All strains examined were isolated clinically from University Hospital Complex, Fez, Morocco. These bacterial and fungal strains were found to be multi-drug-resistant.

#### 3.3.1. Assessment Procedure of the Antimicrobial Capacity

The antibacterial and antifungal performance of *M. longifolia* EO was examined using the disc diffusion technique [73]. Briefly, 15–20 mL of Mueller–Hinton agar (MH) and malt extract (ME) were poured into Petri plates. Inoculant was primed from fresh cultivars grown in the MH and ME environments in (0.9% NaCl) under sterile conditions. Secondarily, 6 mm diameter Whatman paper discs were imbibed with 10 μL of EO and subsequently placed in Petri dishes inoculated with bacteria (10^6^ to 10^8^ CFU/mL) and yeast. Following 24 and 48 h of inoculating Petri plates with bacteria and yeast at 37 °C and 30 °C, respectively, the diameters of inhibition and the percentages of inhibition were measured for the bacterial, fungi, and yeast strains. Antibiotics: streptomycin, erythromycin, and fluconazole. Each experience was executed in triplicate.

#### 3.3.2. Minimum Inhibitory Concentration (MIC)

The microdilution technique is used to calculate the MIC of the EO of *Mentha longifolia* against various microbiological strains [74]. This was accomplished using a dilution series of *M. longifolia* EO (0.097 to 50 mg/mL). One hundred microliters of *M. longifolia* EO with a 1:10 (*v*/*v*) dilution of DMSO (10%) each concentration of such dilution series was mixed in microplate wells with sterile Mueller–Hinton broth and 30 μL of microbial inoculate at a finish microbial concentration of (10^8^ CFU/mL). After an incubation period of 48 h to 7 nights for fungi and 24 h for bacteria at 37 °C [75], the MIC was calculated by the colorimetric mean using resazurin 0.015% [76].

### 3.4. Insecticidal Potential

#### 3.4.1. Breeding of Insects

The glass should be placed with a temperature of 27 ± 1 °C, relative humidity of 70 ± 5%, and a photoperiod of 14 h (light)/10 h (dark). This setting resembles the natural conditions that *Callosobruchus maculatus* is used to, and will promote their development and reproduction.

#### 3.4.2. Toxicity of Essential Oils against *Callosobruchus maculatus*: Fumigation Test

The fumigation test was carried out to evaluate the insecticide activity of the essential oil in the vapor phase. The test was carried out in 1-L jars, and to avoid direct contact with insects, pieces of Whatman No. 1 paper (3 × 3 cm) saturated with various essential oil concentrations of 4 μL, 12 μL, 16 μL, and 20 μL/L of air were affixed to the inside surface of each jar’s cover. Then, 20 adult insects of *C. maculatus* (10 males and 10 females) aged 0 and 48 h were introduced separately into each jar, and the control was performed without treatment. Three replicates were performed for all tests. Dead individuals were counted daily for each dose for one day. Using a magnifying binocular, the egg-laying capability of *C. maculatus* females was estimated. The jars (controls and treated), were kept in a culture chamber set at a temperature of 27 ± 1 °C, a relative humidity of 70.5%, and a photoperiod of 14 h (light)/10 h (darkness), until the emergence phase of adults [77].

Mortality of adults: The observed mortality rate is corrected by the Abbott formula [78].

$$Pc=\frac{Po-Pt}{100-Pt}\times 100$$

where *Pc* = % Corrected mortality, *Po* = the trial’s observed mortality, and *Pt* = mortality observed under control.

#### 3.4.3. Repellent Influence of Essential Oils

The repulsiveness of essential oils to adults of *Callosobruchus maculatus* (*C. maculatus*) has been studied according to the preferred zone methodology used on filter paper with the method described by McDonald et al. [79]. Whatman No. 1 filter paper discs (Diameter 8 cm) were cut into two equal parts, one imprinted with each essential oil tested at different doses of 4 μL, 12 μL, 16 μL, and 20 μL diluted in 0.5 mL of acetone, the other part was imbibed exclusively with the same volume of acetone (Negative control). The two filter paper halves were air-dried and then taped together. Ten adult insects of *C. maculatus* (5 males and 5 females) were placed in the center of the boxes. Three replicates are performed for each dose. A half-hour after each treatment, the number of individuals on each of the two portions was tallied.

The percent repulsion (*PR*) of essential oils for adults of *C. maculatus* was calculated using the formula.

$$PR\left(\%\right)=\frac{N-NT}{N+NT}\times 100$$

*PR* = Percentage repulsion, *N* = Number of individuals present in the acetone portion only. *NT* = Number of individuals present in the part subjected to the essential oil diluted in acetone solvent.

### 3.5. Molecular Docking

To theoretically study the role of the identified EO in *M. longifolia* concerning their potential antioxidant and antimicrobial properties, molecular docking was performed.

#### 3.5.1. Ligand Preparation

To conduct the ligand preparation, the molecules existing in the *M. longifolia* EO were obtained from the PubChem database in SDF format. Subsequently, the LigPrep tool from the Schrödinger Software program (version 11.5) was employed, using the OPLS3 force field, to prepare the ligands. Each ligand was subjected to ionization state selection at pH 7.0 ± 2.0, resulting in the generation of 32 stereoisomers for each molecule [80].

#### 3.5.2. Protein Preparation

The structure of human NADPH oxidase (PDB ID: 2CDU) [81] and the beta-ketoacyl-[acyl carrier protein] synthase from *Escherichia coli* (PDB ID: 1FJ4) [82], *Staphylococcus aureus* nucleoside diphosphate kinase (PDB ID: 3Q8U) [81], a beta-1,4-endoglucanase from *Aspergillus niger* (PDB ID: 5I77) [83], and sterol 14-alpha demethylase (CYP51) from a pathogenic yeast *Candida albicans* (PDB ID: 5FSA) [84] were directly obtained from the Protein Data Bank. The optimization process was completed by adding hydrogen atoms, completing bond orders, removing water molecules, assigning hydrogen bonds, fixing the potential of receptor atoms, and energy minimization using the OPLS3 force field [80].

### 3.6. Statistical Analysis

Average standard deviation values were obtained with GraphPad Prism 8.0.1. The results were compared through a variance analysis (ANOVA) followed by the Tukey test. The difference at *p* < 0.05 was interpreted as significant. The Principal Component Analysis (PCA) was conducted using historical data.

## 4. Conclusions

In the current study, we examined the chemical components, antioxidant, antimicrobial, and insecticidal properties of EO extracted from *M. longifolia* on nosocomial antibiotic-resistant microorganisms and stored grain pests. Our findings indicated that *Mentha longifolia* L. EO is rich in terpene compounds with a wide range of biological activities including antioxidant, antibacterial, antifungal, and insecticidal potentials against bacteria, yeast, and insect pests. This provides the potentiality of using *M. longifolia* essential oil and its active components in several biological applications, including the creation of new products in the pharmaceutical industry, and as a preservative agent of fresh food in the food industry instead of chemical preservatives.

This study can be reinforced by more in-depth research, including quantifying numerous new compounds, as well as purifying and molecularly identifying the chemical structures that underlie particular biological properties. The safety of the studied oils needs to be studied using preclinical tests on non-human models before any potential medical use.

## Figures and Tables

**Table 1:** Phytochemical constituents contained in the EO extracted from Moroccan *M. longifolia*.

Peak	RT (min)	Compounds	Chemical Classes	RI		Molecular Formula	Area%
				Cal	Lit		
1	18.206	Bornyl acetate	MO	1277	1286	C_12_H_20_O_2_	4.47
2	20.249	Piperitenone oxide	MO	1236	1366	C_10_H_14_O_2_	53.43
3	21.899	Caryophyllene	ST	1494	1432	C_15_H_24_	20.02
4	22.959	cis-Muurola-4(15),5-diene	ST	1435	1467	C_15_H_24_	1.87
5	23.435	(−) Germacrene D	ST	1515	1482	C_15_H_24_	16.53
6	23.797	Bicyclogermacrene	ST	1499	1495	C_15_H_24_	1.93
7	28.221	α-Cadinol	ST	1580	1654	C_15_H_26_O	1.75
			Chemical classes				
			Monoterpene (MO)				
			Sesquiterpene (ST)				
Total (%)							100.00

**Table 2:** Antioxidant activities of *M. longifolia* essential oil.

	ML-EO	BHT	TROLOX	AA
DPPH IC_50_ (μg/mL)	1.49 ± 0.00 ^a^	42 ± 0.01 ^b^	-	-
ABTS IC_50_ (μg/mL)	0.051 ± 0.06 ^a^	-	24.14 ± 0.19 ^b^	-
RP EC_50_ (μg/mL)	0.80 ± 0.01 ^a^	-	-	31 ± 0.07 ^b^
TAC (mg/mL)	315.53 ± 0.01	-	-	-

Column values with the same letters do not present significant differences (*p* > 0.05, inside the sample under study); DPPH: 2,2-Diphenyl-1-picrylhydrazyl test; ABTS: 2,2′ -azino-bis (3-ethylbenzothiazoline-6-sulfonic acid) assay; RP: Reducing Power test; TAC: Total antioxidant capacity; BHT: Butylated hydroxytoluene; AA: Ascorbic acid.

**Table 3:** Diameter of the inhibition zone (mm), and Minimum inhibitory concentration (MIC in µg/mL) from EO of *M. longifolia* in comparison with antibiotics.

Simple		Gram-Negative Bacteria		Gram-Positive Bacteria	
		*P. aeruginosa*	*E. coli*	*S. aureus*	*B. subtilis*
Essential oil	Antibacterial activity (mm)	24.50 ± 0.71	7.55 ± 0.64	7.50 ± 0.71	16.00 ± 1.41
	MIC (µg/mL)	0.005 ± 0.00	0.010 ± 0.00	0.011 ± 0.00	0.015 ± 0.00
Streptomycin	Antibacterial activity (mm)	6 ± 0.00 (R)	6 ± 0.00 (R)	11 ± 0.01	R
	MIC (µg/mL)	-	-	1.55 ± 0.00	-
Erythromycin	Antibacterial activity (mm)	6 ± 0.00 (R)	6 ± 0.00 (R)	6 ± 0.00 (R)	6 ± 0.00 (R)
	MIC (µg/mL)	-	-	-	-
DMSO (10%)	Antibacterial activity (mm)	6 ± 0.00 (R)	6 ± 0.00 (R)	6 ± 0.00 (R)	6 ± 0.00 (R)

R: Resistant. MIC: minimum inhibitory concentration.

**Table 4:** Results of the antifungal ability of the EO of *M. longifolia* diameter of the inhibition zone (mm) and MIC (MIC in mg/mL).

Simple	Antifungal Activity by Disc Method (mm)		Antifungal Activity by the Microdilution Method (MIC in mg/mL)	
	*Candida albicans*	*Aspergillus niger* (%)	*Candida albicans*	*Aspergillus niger*
Essential oil	20.00 ± 0.01	1.50 ± 0.02	0.005 ± 0.00	0.005 ± 0.00
Fluconazole	6 ± 0.00 (R)	8.25± 1.02	-	7.15 ± 0.01
DMSO	6 ± 0.00 (R)	6 ± 0.00 (R)	6 ± 0.00 (R)	6 ± 0.00 (R)

R: Resistant.

**Table 5:** Effect of ML-EO on *C. maculatus* mortality as a function of concentration and exposure durations based on fumigation test.

Essential Oil	Doses of EO (µL/L of Air/10 g)	Exposure Time (Hours)				
		3 h	6 h	12 h	18 h	24 h
ML-EO	Control	0 ± 0.00	0 ± 0	0 ± 0	0 ± 0	0 ± 0
	4	0 ± 0.00	44.66 ± 9.23	66.66 ± 2.88	93.33 ± 7.63	100 ± 0
	12	3.33 ± 2.88	55 ± 10	71.66 ± 5.77	95 ± 8.66	100 ± 0
	16	11.66 ± 7.63	61.66 ± 2.88	86.66 ± 8.00	100 ± 0.00	100 ± 0
	20	28.33 ± 5.77	63.33 ± 8.66	91.66 ± 2.33	100 ± 0.00	100 ± 0.00

**Table 6:** Lethal concentration, and chi-square (χ^2^) values of *M. longifolia* and *M. aquatica* essential oil tested by fumigation assay on *C. maculatus*.

	Treatment (h)	LC_50_ (μL/L)	95% CI	LC_90_ (μL/L)	95%CI	df	χ^2^
ML-EO	3	29.42	19.96–309.41	73.09	34.92–13,080.778	2	0.098
	6	5.62	-	18.74	-	2	
	12	2.24	0.0–4.68	18.71	10.52–139,889.51	2	0.624
	18	0.75	-	3.47	-	2	0.914
	24	-	-	-	-	-	-

(-): data are absent since the insects were killed within the experiment’s first minute.

**Table 7:** Effect of EOs on different biological parameters of *C. maculatus* (mean ± SD).

Dose (μL/L Air)	Fecundity	Fertility (%)	Adult Emergence (%)
	ML-EO	ML-EO	ML-EO
4 µL	21 ± 2.65	80 ± 5	47.82 ± 4.18
12 µL	7.34 ±2.51	22.11± 3.64	0 ± 0
16 µL	0 ± 0	0 ± 0	0 ± 0
20 µL	0 ± 0	0 ± 0	0 ± 0
Control	196.67 ± 11.55	94.02 ± 11.55	93.35 ± 5.20

**Table 8:** Repellent index of the ML-EO against *C. maculatus*.

Dose (μL/cm^2^)	RI (Mean ± SD)	Repellency Class	The Average Rate of Repulsively (%)
	ML-EO		ML-EO
4	77 ± 5.00	Repellent	89.75
12	90 ± 8.16	Repellent	
16	92 ± 5.00	Repellent	
20	100 ± 0.00	Repellent	

**Table 9:** Docking results in ligands in different receptors.

	Antioxidant Activity	Antibacterial Activity		Antifungal Activity	
Title	2CDU	1FJ4	3Q8U	5FSA	5I77
	Glide Gscore (Kcal/mol)				
(−) Germacrene D	−5.085	−5.998	−4.128	−6.825	−4.054
α-Cadinol	−6.041	−5.467	−5.714	−7.182	−4.377
Bicyclogermacrene	−4.576	−5.369	−4.211	−7.085	−4.187
Bornyl acetate	−3.585	−5.436	−3.389	−5.497	−4.022
Caryophyllene	−3.948	−5.064	−4.157	−7.025	−3.582
cis-Muurola-4(15), 5-diene	−4.811	−6.321	−4.867	−7.486	−3.908
Piperitenone oxide	−5.195	−7.104	−4.771	−5.606	−4.687

## Data Availability

Data will be made available on request.
